# Vasopressor Requirements after Initiation of Venovenous Extracorporeal Membrane Oxygenation in Patients with Severe Respiratory Failure

**DOI:** 10.1016/j.aicoj.2025.100023

**Published:** 2026-01-16

**Authors:** Bernhard Nagler, Nina Buchtele, Peter Schellongowski, Oliver Robak, Alexander Hermann, Thomas Staudinger

**Affiliations:** Medical University of Vienna, Department of Medicine I, Intensive Care Unit 13i2, Währinger Gürtel 18-20, 1090 Vienna, Austria

**Keywords:** Acute cor pulmonale (ACP), Acute respiratory distress syndrome (ARDS), Extracorporeal membrane oxygenation (ECMO), Haemodynamics, Heart-lung interactions, Respiratory acidosis, Right ventricular function, Vasoactive-Inotropic Score (VIS), Vasopressors

## Abstract

**Background:**

Patients with severe respiratory failure frequently suffer from concomitant haemodynamic compromise. By correcting respiratory acidosis and permitting reduced mechanical ventilation pressures, venovenous extracorporeal membrane oxygenation (V-V ECMO) may indirectly improve haemodynamics. The aim of this study was to assess how vasopressor requirements changed after V-V ECMO cannulation, and which factors were the primary drivers of this change.

**Methods:**

This retrospective single-centre study included 107 consecutive adult recipients of V-V ECMO from 2010 to 2024 who required noradrenaline within 24 h before ECMO cannulation. The primary outcome was the change in Vasoactive-Inotropic Score (VIS) from Day 0 (24 h before) to Day 1 (24 h after ECMO initiation). Secondary outcomes included changes in fluid balance, ventilator settings, blood gas and laboratory parameters. A linear mixed-effects model was used to assess the effects of daily net fluid balance, mean airway pressure (P_aw_), mean daily pH, arterial partial pressure of oxygen (PaO_2_), arterial partial pressure of carbon dioxide (PaCO_2_), mean daily propofol dose, and lactate on the VIS over time (“Day −2” to “Day +3”).

**Results:**

From Day 0 to Day 1, the daily mean VIS significantly decreased from a median of 14 (IQR 6, 30) to a median of 12 (5, 22). This was accompanied by significant reductions in P_aw_ and PaCO₂, and a significant increase in arterial pH (p < 0.001 for all). In the multivariate model, a higher arterial pH was significantly associated with a lower VIS (β = −9.2 per +0.1-unit, p < 0.001). Higher lactate was associated with higher VIS (β = 4.5, p < 0.001). Sensitivity analyses revealed more pronounced effects of pH increase on VIS reduction in patients with high noradrenaline requirements.

**Conclusions:**

After initiation of V-V ECMO, a significant decrease in vasopressor requirements was observed, this benefit being directly attributable to the correction of severe respiratory acidosis.

## Introduction

Patients with acute respiratory distress syndrome (ARDS) commonly suffer from haemodynamic compromise [1]. The pathophysiology of ARDS entails pulmonary vascular dysfunction, leading to pulmonary hypertension [2]. The subsequent increase in right ventricular (RV) afterload can result in RV dysfunction. This complication occurs in approximately 22–25% of patients with ARDS even under protective ventilation strategies [1,3]. RV dysfunction ranges from echocardiographic evidence of acute cor pulmonale (ACP) to overt RV failure resulting in shock (7% of patients with ARDS [1]), which is associated with significantly increased mortality [1]. These effects are further amplified by the negative hemodynamic consequences of high mechanical ventilation pressures, which are required to maintain adequate pulmonary gas exchange [2,4,5]. Alongside RV failure, sepsis - the most frequent underlying condition of ARDS [6] - can be a major contributor to haemodynamic compromise. Elevated vasopressor requirements may be a consequence of vasopressor resistance, which can be a result of metabolic derangements including acidosis, electrolyte derangements, or inflammatory mediators, all of which are likely to be encountered in this group of severely ill patients [7].

Venovenous extracorporeal membrane oxygenation (V-V ECMO) has become an important therapeutic option in patients with severe ARDS [8]. V-V ECMO can indirectly reduce pulmonary vascular resistance by reversing hypoxic and hypercapnic pulmonary vasoconstriction and by allowing for lower mechanical ventilation pressures [[9], [10], [11], [12], [13]]. Conversely, an excessive reduction in mechanical ventilation pressures facilitated by ECMO carries the risk of pulmonary atelectasis, which may increase PVR by shifting lung volume to the lower limb of the PVR-inflation curve [14]. In a recent study, Odish et al. described that vasopressor requirements significantly decreased 24 h after V-V ECMO initiation [15]. However, potential confounders, such as fluid administration and changes in sedative agents, were not regarded.

The current study examined the net effect of initiating V-V ECMO therapy on vasopressor requirements, while accounting for concurrent changes in blood gases, mechanical ventilation, sedation, and fluid balance. These findings may inform clinical decision-making for patients with ARDS and hemodynamic compromise and guide hemodynamic management around ECMO cannulation.

## Study design and methods

### Patients and study design

This single-centre retrospective cohort study was conducted at a tertiary referral hospital's medical intensive care unit. It included consecutive adult patients who received V-V ECMO for respiratory support between 01 January 2010 and 30 June 2024. Patients were excluded if they did not receive noradrenaline or if their ECMO initiation occurred at a different institution before transfer to the study centre. Data for the study were obtained from the electronic patient data management system and the local ECMO registry. The institutional review board of the Medical University of Vienna, Austria, approved the study (Ref. 1630/2024).

Study time periods were defined in 24-h intervals relative to the time of ECMO initiation (t = 0). “Day 0” corresponded to the 24 h preceding ECMO initiation (t = −24 h to t = 0), "Day 1" to the period from t = 0 to t = 24 h, and "Day -1" from t = −48 h to t = −24 h. This pattern was applied to all subsequent study days. The daily net fluid balance was recalculated to align with the defined study days.

Primary endpoint was the change in mean daily Vasoactive-Inotropic Score (VIS) between “Day 0” and “Day 1”. For this study, continuous infusions of noradrenaline, dobutamine, and vasopressin were regarded. VIS was calculated as 100 × noradrenaline dose (µg/kg/min) + dobutamine dose (µg/kg/min) + 10,000 × vasopressin dose (U/kg/min) [16]. Other medication components of VIS or its commonly used extensions, such as adrenaline, dopamine, phenylephrine, and milrinone, were not included as they were not administered in the study population.

Secondary endpoints included changes of fluid balance, ventilator settings, blood gas parameters and N-terminal prohormone of brain natriuretic peptide (NT-proBNP) between “Day 0” and “Day 1”. Another secondary endpoint was the effect of the following parameters on the VIS over time (“Day −2” to “Day +3”): daily net fluid balance, mean airway pressure (Paw), mean daily arterial pH, arterial partial pressure of oxygen (PaO₂), arterial partial pressure of carbon dioxide (PaCO₂), and mean daily propofol dose. For pressure control ventilation, P_aw_ was estimated as P_aw_ = (PIP − PEEP) × (T_I_/T_tot_) + PEEP, where PIP is peak inspiratory pressure, PEEP is positive end-expiratory pressure, T_I_ is inspiratory time, and T_tot_ is total respiratory cycle time [17]. For biphasic positive airway pressure ventilation (BIPAP/Bi-Vent), P_aw_ was estimated as P_aw_ = (P_high_ × T_high_ + P_low_ × T_low_)/(T_high_ + T_low_), where P_high_ is upper pressure level, P_low_ is lower pressure level, T_high_ is the duration of P_high_, and T_low_ is the duration of P_low_ [18]. Plateau pressure and derived driving pressure measurements were excluded from the analysis due to the lack of routinely documented ventilatory hold manoeuvres.

## Local treatment practice

Indications for V-V ECMO included severe refractory hypoxemia, hypercapnia, or bridging to lung transplantation. Management of ECMO, mechanical ventilation, sedation, fluid administration and catecholamine therapy was guided by institutional standards without a rigid protocol. Patients were ventilated using pressure-controlled or biphasic positive airway pressure modes, with positive end-expiratory pressure (PEEP) titrated individually based on patient physiology.

## Statistical analysis

All analyses were performed in R 4.5.1 (The R Foundation for Statistical Computing, Vienna, Austria). A P-value <0.05 was considered statistically significant.

For descriptive analysis, demographic and baseline data are presented as medians and interquartile ranges (IQR). Missing data for medication, ventilation, and fluid balance were assumed to be missing-at-random (MAR). A comprehensive audit of missing data per variable and time point is provided in e-Table 1. Given the likelihood-based approach of the linear mixed-effects model, no explicit imputation was performed.

To provide a descriptive overview of the immediate clinical response, the change in VIS from “Day 0” to “Day 1” was assessed using the Wilcoxon signed-rank test.

To account for longitudinal data structure, within-patient variability, and time-varying covariates across the full observation period (Day −2 to +3), a linear mixed-effects model was utilised to analyse the changes in VIS over time. The final model included VIS as the dependent variable with time (as a categorical factor), daily fluid balance, P_aw_, pH, PaO_2_, PaCO_2_, propofol dose, lactate (each daily mean), and renal replacement therapy as fixed effects. To account for inter-individual variability, a random intercept was included for each patient. To facilitate clinical interpretation of the model estimates, continuous predictor variables were rescaled prior to analysis. Adjusted mean differences (Beta coefficients) are reported corresponding to a 0.1-unit increase for arterial pH, a 10-mmHg increase for PaCO₂, and a 1,000-mL increase for daily net fluid balance. In an additional exploratory analysis, all continuous predictor variables were standardised (z-score scaled) to a mean of 0 and a standard deviation of 1 to allow for a more direct comparison of the relative impact of each predictor on VIS. While other sedative agents with known haemodynamic effects, such as midazolam and dexmedetomidine, were administered, only the mean daily propofol dose was included as a fixed effect in the model to maintain statistical power and avoid overfitting. Propofol was selected as it was the most frequently used sedative in our cohort (n = 90 on Day 0) and is considered to have the most significant hemodynamic impact. In contrast, midazolam was used less frequently (n = 53 on Day 0), and dexmedetomidine was used too infrequently (n = 4 on Day 0) to be included in the model. Model performance and assumptions were verified using standard diagnostic plots and performance indices [19]. Multicollinearity among fixed effects was assessed using Variance Inflation Factors (VIFs), with values <5 considered indicative of no significant collinearity. VIF was calculated and plotted (e-Figure 1) using the package “performance” in R [20]. Estimated marginal means (EMMs) were calculated using the *emmeans* package in R [21] to assess the predicted VIS at specific time points relative to ECMO initiation. Pairwise comparisons between these estimated means were performed, with p-values adjusted using the Tukey method to control for the family-wise error rate arising from multiple comparisons. An additional model was fit using a time-lagged covariable for fluid balance to further explore the association between fluid balance and VIS.

Additionally, sensitivity analyses were performed by repeating the analyses for the subgroups of patients requiring a mean noradrenaline dose ≥0.2 µg/kg/min on “Day 0”, and patients with noradrenaline ≤0.05 µg/kg/min.

## Results

### Baseline characteristics

From a cohort of 136 consecutive patients undergoing V-V ECMO initiation at the study site, 107 required noradrenaline within 24 h before ECMO start and were included in the final analysis. Baseline parameters are presented in Table 1. The cohort had a median age of 50 years (IQR 41, 61), 63% were male, and median Body Mass Index was 27 kg/m² (23, 32). Median Sequential Organ Failure Assessment Score (SOFA) on admission was 9.0 (8.0, 12.0), median lowest PaO_2_/F_I_O_2_ before ECMO was 71 mmHg (58, 93), lowest pH 7.25 (7.17, 7.31), and P_aw_ 18.3 mbar (15.5, 21.3). The predominant causes of respiratory failure were Coronavirus disease 2019 (COVID-19) (38%) and influenza (16%).

**Table 1:** Baseline characteristics.

Characteristic	N = 107^1^
Sex	
Female	40 (37%)
Male	67 (63%)
Age at admission (years)	50 (41, 61)
Height (cm)	173 (167, 180)
Weight (kg)	85 (70, 100)
Body Mass Index (kg/m²)	27 (23, 32)
SOFA (Sequential Organ Failure Assessment) score at ICU Admission	9.0 (8.0, 12.0)
SAPS III (Simplified Acute Physiology Score III) at ICU admission	66 (61, 74)
Lowest PaO₂/FiO₂ ratio on day of ECMO start (mmHg)	71 (58, 93)
Lowest pH on day of ECMO start	7.25 (7.17, 7.31)
Lowest PaO₂ on day of ECMO start (mmHg)	61 (55, 68)
Highest PaCO₂ on day of ECMO start (mmHg)	77 (65, 91)
Mean Peak Inspiratory Pressure on day of ECMO start (mbar)	30.5 (27.6, 33.3)
Mean PEEP on day of ECMO start (mbar)	12.0 (8.7, 15.0)
Mean Respiratory Rate on day of ECMO start (breaths/min)	20.6 (17.5, 23.8)
Mean Airway Pressure on day of ECMO start (mbar)	18.3 (15.5, 21.3)
NT-proBNP at ECMO start (pg/mL)	548 (229, 2,824)
Noradrenaline at ECMO start (No. of patients)	107 (100%)
Mean Noradrenaline Dose on day of ECMO start (µg/kg/min)	0.14 (0.06, 0.29)
Dobutamine at ECMO start (No. of patients)	7 (6.5%)
Vasopressin at ECMO start (No. of patients)	15 (14%)
Mean Vasoactive-Inotropic Score on day of ECMO start	14 (6, 30)
Highest Lactate on day of ECMO start (mmol/L)	1.50 (1.10, 2.90)
Cause of respiratory failure	
COVID 19	41 (38%)
Influenza	17 (16%)
Exacerbation of other chronic respiratory disease	14 (13%)
Other infectious pneumonia	12 (11%)
COPD/Asthma	5 (4.7%)
Haematological disorder	5 (4.7%)
Other	13 (12.1%)
Renal Replacement Therapy During ICU Stay	33 (31%)
Duration of ECMO support (days)	15 (8, 30)
ICU Survival	70 (65%)
Hospital Survival	66 (63%)

ECMO, extracorporeal membrane oxygenation; FiO₂, fraction of inspired oxygen; ICU, intensive care unit; IQR, interquartile range; NT-proBNP, N-terminal pro–B-type natriuretic peptide; PaCO₂, partial pressure of arterial carbon dioxide; PaO₂, partial pressure of arterial oxygen; PEEP, positive end-expiratory pressure; SAPS III, Simplified Acute Physiology Score III; SOFA, Sequential Organ Failure Assessment; VIS, Vasoactive-Inotropic Score.

1 n (%); Median (Q1, Q3).

As per inclusion criteria, all patients received noradrenaline before ECMO start, with a median of the mean dose of noradrenaline on “Day 0” of 0.14 µg/kg/min (0.06, 0.29). Before ECMO start, 7 (6.5%) patients received dobutamine, 15 (14%) received vasopressin, and median VIS was 14 (6, 30). No continuous infusions of other vasopressors were administered during the regarded study days. 33 (31%) patients received renal replacement therapy at any time during their ICU stay.

The median duration of ECMO support was 15 days (8, 30), and hospital survival was 63%.

### Primary endpoint

**Fig. 1:**
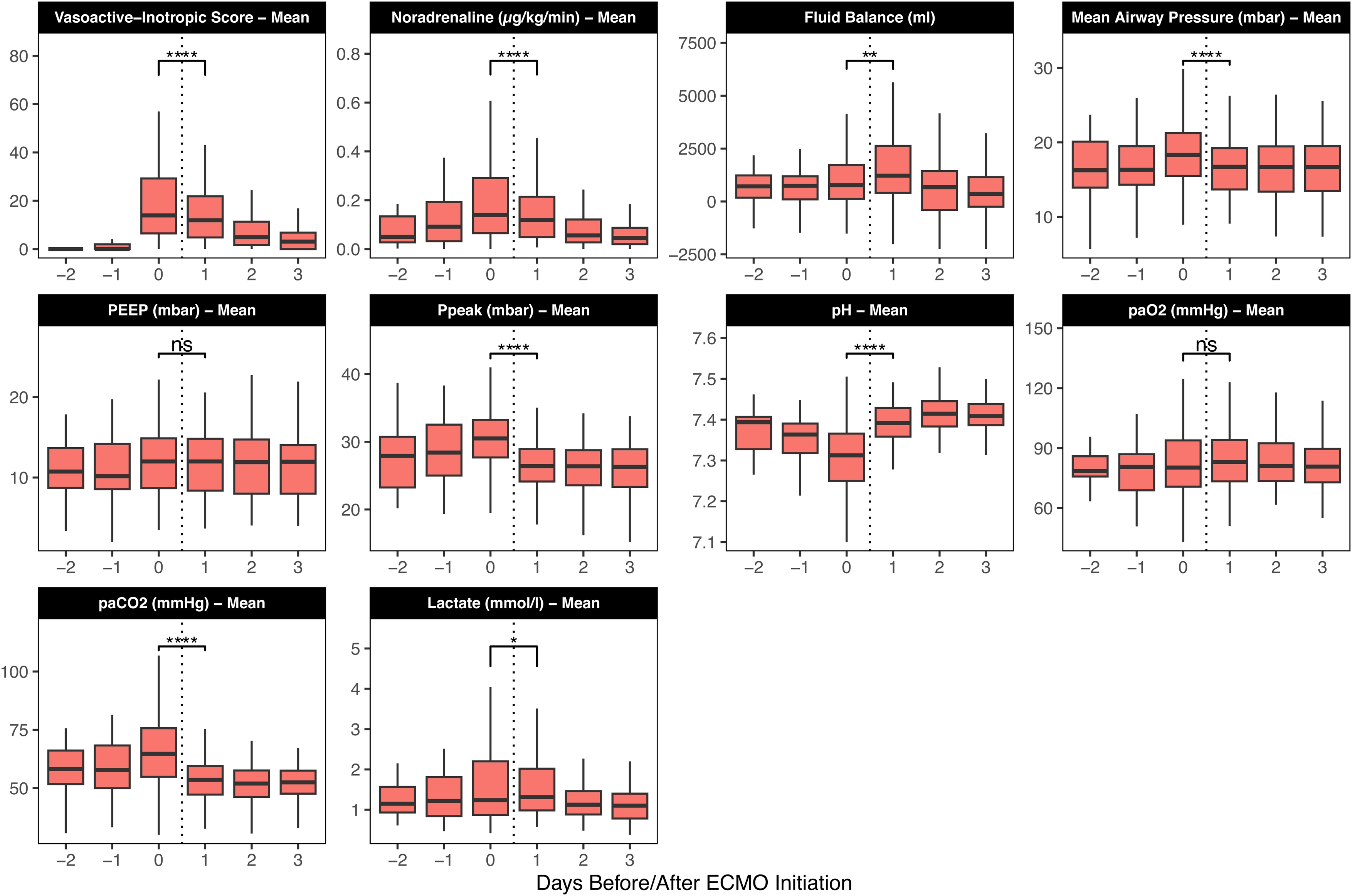
Changes in Haemodynamic, Laboratory, and Ventilation Parameters Around ECMO Initiation. Boxplots illustrate the change in key clinical parameters before and after ECMO start. Days were defined using the following scheme: Day 0 - the 24 h immediately preceding ECMO start, Day 1 - the first 24 h after ECMO start, etc. The central line in each box represents the median value, the box boundaries indicate the interquartile range (IQR), and the whiskers extend to the most extreme data point which is no more than 1.5 times the IQR from the box. P-values, calculated using the Wilcoxon signed-rank test, are denoted as follows: ns p > 0.05; *p ≤ 0.05; **p ≤ 0.01; ***p ≤ 0.001; ****p ≤ 0.0001. ECMO, extracorporeal membrane oxygenation; PaCO₂, partial pressure of arterial carbon dioxide; PaO₂, partial pressure of arterial oxygen; PEEP, positive end-expiratory pressure; Ppeak, peak inspiratory pressure.

Changes of mechanical ventilation, haemodynamic and laboratory parameters are provided in Table 2, e-Table 2 and visually summarised in Fig. 1. Descriptive comparison of the immediate peri-cannulation period (Table 2) showed that daily mean VIS significantly decreased between “Day 0” (24 h before ECMO start) and “Day 1” (24 h after ECMO start) from a median of 14 (6, 30) to a median of 12 (5, 22).

**Table 2:** Descriptive comparison of clinical parameters between Day 0 (24 h before ECMO start) and Day 1 (24 h after ECMO start).

Characteristic	Day 0 N = 107^1^	Day 1 N = 107^1^	p-value^2^
Mean Vasoactive-Inotropic Score	14 (6, 30)	12 (5, 22)	<0.001
Mean Noradrenaline Dose (µg/kg/min)	0.14 (0.06, 0.29) (N = 107)	0.12 (0.05, 0.21) (N = 106)	<0.001
Cumulative Fluid Balance (Day 0−1) (mL)		+2,054 (+791, +3,844)	
Mean Mean Arterial Blood Pressure (mmHg)	78 (72, 82)	80 (75, 85)	0.002
Mean Heart Rate (bpm)	97 (86, 109)	89 (77, 97)	<0.001
Mean NT-proBNP (pg/mL)	548 (229, 2,824)	795 (338, 2,612)	0.3
Mean Lactate (mmol/L)	1.24 (0.87, 2.20)	1.31 (0.98, 2.03)	0.010
Mean pH	7.31 (7.25, 7.37)	7.39 (7.36, 7.43)	<0.001
Mean HCO3- (mmol/L)	29 (25, 32)	31 (27, 35)	<0.001
Mean SpO_2_ (%)	93.7 (92.4, 95.6)	96.4 (95.0, 98.1)	<0.001
Mean PaO₂ (mmHg)	80 (71, 94)	83 (73, 94)	0.5
Mean PaCO₂ (mmHg)	65 (55, 76)	54 (47, 60)	<0.001
Mean Mean Airway Pressure (mbar)	18.3 (15.5, 21.3)	16.7 (13.7, 19.2)	<0.001
Mean PEEP (mbar)	12.0 (8.7, 15.0)	12.0 (8.4, 14.8)	0.13
Mean Peak Inspiratory Pressure (mbar)	30.5 (27.6, 33.3)	26.4 (24.1, 28.9)	<0.001
Mean Tidal Volume (mL)	410 (331, 492)	261 (209, 344)	<0.001
Mean Respiratory Rate (breaths/min)	20.6 (17.5, 23.8)	13.7 (12.2, 16.0)	<0.001
Mean Propofol Dose (mg/kg/hr)	3 (2, 4) (N = 90)	3 (2, 3) (N = 83)	0.024
Leukocyte Count (G/L)	14 (10, 19)	11 (9, 15)	0.002
CRP (mg/dL)	16 (8, 25)	22 (15, 29)	0.2
Serum Creatinine (mg/dL)	0.78 (0.58, 1.03)	0.84 (0.60, 1.06)	0.092
Renal Replacement Therapy	7 (6.5%)	13 (12%)	0.2

1 Median (Q1, Q3).

2 Wilcoxon signed rank test with continuity correction; Wilcoxon signed rank exact test bpm, beats per minute; HCO3-, Plasma Bicarbonate; N, number of patients; NT-proBNP, N-terminal pro–B-type natriuretic peptide; PaCO₂, partial pressure of arterial carbon dioxide; PaO₂, partial pressure of arterial oxygen; PEEP, positive end-expiratory pressure; SpO_2_, peripheral oxygen saturation; SOFA, Sequential Organ Failure Assessment.

### Secondary endpoints

From “Day 0” to “Day 1”, mean daily noradrenaline dose decreased from a median of 0.14 (0.06, 0.29) to 0.12 (0.05, 0.21) µg/kg/min (p < 0.001) (Table 2). Similarly, mean arterial pressure increased and heart rate decreased significantly, while median cumulative fluid balance across the two days was +2,054 (+791, +3,844) mL (Table 2). Regarding ventilation parameters, mean daily P_aw_ and PaCO_2_ decreased, mean arterial pH increased, while mean PaO_2_ remained stable (Table 2). Mean daily propofol dose slightly changed and remifentanil dose increased, whereas doses of sufentanil, midazolam, dexmedetomidine, and vasopressin remained constant (Table 2 & e-Table 2).

**Fig. 2:**
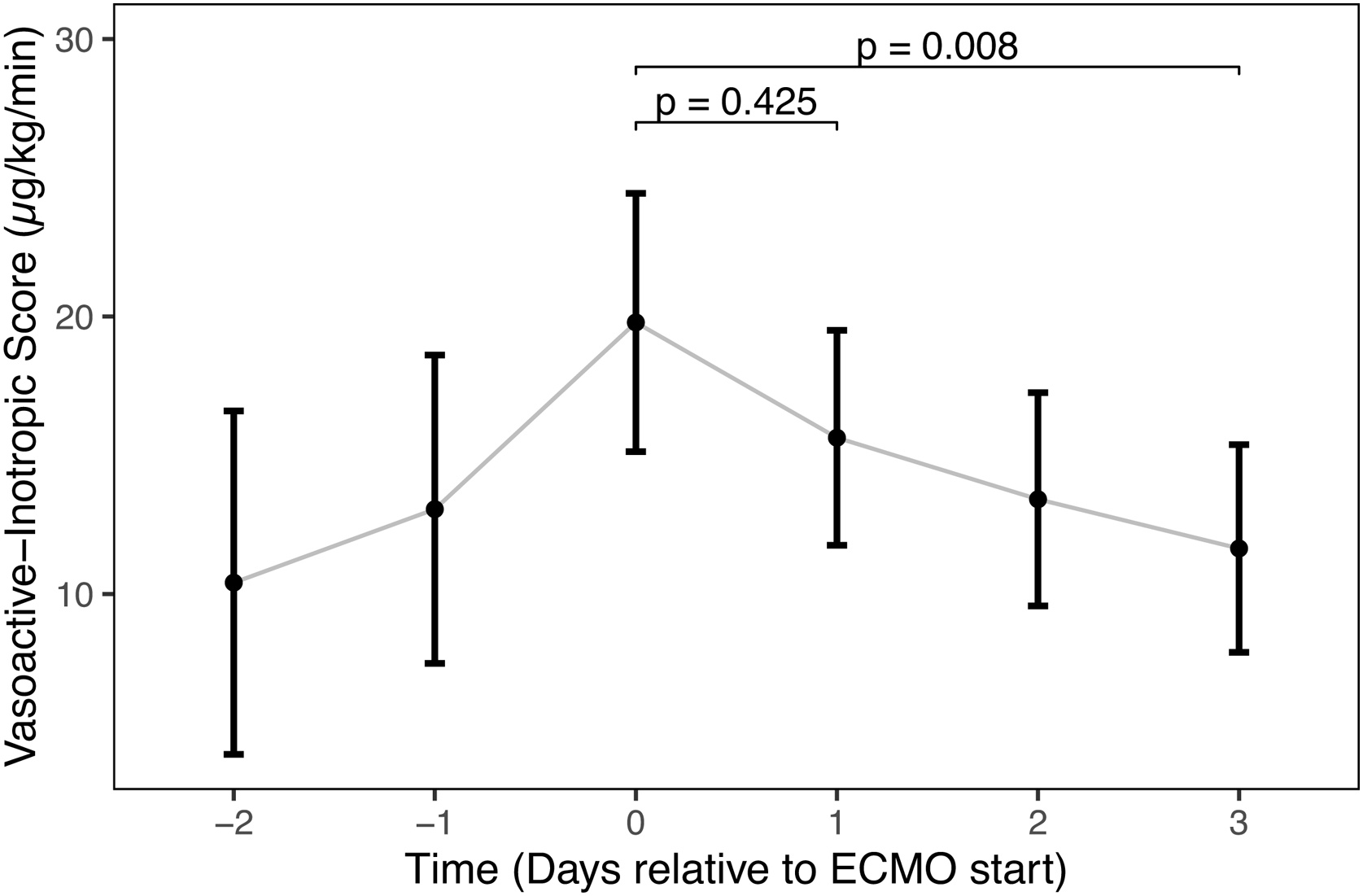
Estimated Marginal Means for Vasoactive-Inotropic Score (VIS) Over Time. The plot displays the estimated marginal means (EMMs) for VIS derived from the linear mixed-effects model, adjusted for all covariates. The points represent the estimated mean VIS at each time point from Day -2 (48 h before ECMO start) to Day 3 (72 h after ECMO start). The vertical error bars indicate the 95% confidence intervals for each estimate. P-values for pairwise comparisons were adjusted using the Tukey method.

A linear mixed-effects model was used to assess factors influencing the mean daily VIS. The model included a random intercept for each patient (n = 107), with a between-patient standard deviation of 9.5. The final multivariable model demonstrated strong goodness-of-fit, with fixed effects explaining half of the total variance in VIS (Marginal R² = 0.50). The full model, accounting for inter-individual variability, explained 69% of the variance (Conditional R² = 0.69). As shown in Table 3, after adjusting for all covariates, a higher arterial pH was significantly associated with a lower VIS. VIS significantly increased with daily net fluid balance and lactate levels doses. Covariate coefficients of a model using standardised predictor variables are provided in e-Table 3. To assess the change in vasopressor requirements over time while accounting for covariates, the estimated marginal means (EMMs) for VIS from the linear mixed-effects model (Fig. 2) were analysed. The analysis revealed that the estimated mean VIS peaked at Day 0 (EMM = 19.8, 95% CI [15.1, 24.4]) before subsequently decreasing. Post-hoc pairwise testing with Tukey's p-value adjustment confirmed that VIS at Day 0 was significantly higher than at Day 3 (mean difference 8.1 points, p = 0.008). No other pairwise comparisons between time points were statistically significant.

**Table 3:** Linear Mixed-Effects Model of Covariates Associated with Vasoactive-Inotropic Score (VIS) from Day -2 to Day 3.

Characteristic	Beta	95% CI	p-value
(Intercept)	691	434, 948	<0.001
Daily Net Fluid Balance (per +1,000 mL)	1.1	0.31, 1.8	0.006
Mean Airway Pressure (per +1 mbar)	0.21	−0.24, 0.66	0.4
Mean Arterial pH (per +0.1 unit)	−9.2	−13, −5.8	<0.001
Mean PaO₂ (per +1 mmHg)	0.02	−0.07, 0.10	0.7
Mean PaCO₂ (per +10 mmHg)	−2.6	−4.5, −0.72	0.007
Mean Propofol Dose (mg/kg/h)	0.01	−0.01, 0.04	0.3
Mean Lactate (mmol/L)	4.5	3.6, 5.4	<0.001
Renal Replacement Therapy (Active)	−2.8	−8.8, 3.2	0.4

CI, Confidence Interval; PaCO₂, partial pressure of arterial carbon dioxide; PaO₂, partial pressure of arterial oxygen.

Conditional R^2^: 0.69; Marginal R²: 0.50.

Note: Estimates represent the change in VIS associated with the specified unit increase in each predictor. Time (study day), which was included as a factor in the model to account for temporal trends, is not shown here for clarity.

### Sensitivity analyses

To further explore the modelled trend towards higher VIS with increased fluid balance, another linear mixed-effects model including a time-lagged fluid balance covariate was fit: daily net fluid balance from the previous day had no significant effect on mean VIS (e-Table 4).

A subgroup analysis of 40 patients requiring a mean noradrenaline dose of ≥0.2 µg/kg/min on Day 0 revealed similar effects (baseline characteristics are provided in e-Table 5): from Day 0 to Day 1, mean VIS, noradrenaline dose and PaCO_2_ decreased, whereas mean pH increased. Median cumulative fluid balance across Day 0 and Day 1 was +2904 (+1726, +5779) mL (e-Table 6). A linear mixed-effects model (n = 40) analogous to the main analysis, showed that mean arterial pH and mean lactate were significantly associated with a lower mean VIS (e-Table 7). Mean VIS estimated by EMMs of the model revealed a peak at Day 0 (EMM = 46.2 (35.6, 56.7)) with a significant decrease from Day 0 to Day 3 (mean difference = 28.1 points, p < 0.001) (e-Figure 2). Finally, a mirrored subgroup analysis was performed on patients with minimal baseline noradrenaline requirements (mean ≤0.05 µg/kg/min on Day “0”; n = 20). In this subgroup, no significant association was found between arterial pH and VIS (p = 0.6, e-Table 8), or any other covariable.

## Discussion

In this retrospective study, the initiation of V-V ECMO was associated with a significant haemodynamic improvement, as reflected by a marked decrease in VIS. Multivariate analysis identified increasing arterial pH as the most powerful predictor of VIS reduction. These observations held true for a subgroup of patients with more severe haemodynamic compromise (mean noradrenaline dose ≥0.2 µg/kg/min during the 24 h before ECMO start), who experienced an even more pronounced decrease of VIS.

These findings align with physiological assumptions and observations from previous studies concerning the haemodynamic consequences of ARDS and the impact of V-V ECMO initiation [[9], [10], [11], [12], [13],^15^]. The development of ACP can contribute to haemodynamic instability in ARDS. It is driven by a pronounced increase in pulmonary vascular resistance (PVR) and a subsequent rise in RV afterload [2]. The key drivers of this detrimental rise in PVR are multifactorial, but opinions vary on which potentially modifiable component exerts the greatest influence on RV afterload [4]. In a multivariate analysis by Vieillard-Baron et al., PaCO_2_ was the only significant predictor of ACP [3]. On the other hand, the effect of mechanical ventilation pressures, such as PEEP and driving pressure, is more intricate due to the U-shaped relationship between lung distension and PVR [2,14]. By correcting respiratory acidosis and enabling a reduction of mechanical ventilation settings, V-V ECMO is uniquely positioned to intervene directly at each of these critical points.

In the primary analysis, we observed a statistically significant decrease in the median observed VIS from 14 (6, 30) on Day 0–12 (5, 22) on Day 1 (p < 0.001), reflecting the raw, unadjusted improvement in vasopressor needs after ECMO initiation. However, our multivariate analysis reveals a more nuanced picture: the reduction in VIS was not an independent effect of time, but directly attributable to the concurrent improvements in the patients' underlying physiological state. Importantly, the association between arterial pH and vasopressor requirements persisted after adjusting for serum lactate and renal replacement therapy. This suggests that the haemodynamic benefit of V-V ECMO is not merely a reflection of general metabolic recovery — indeed, lactate levels slightly increased during the first 24 h — but is directly attributable to the correction of respiratory acidosis (Table 3). To investigate the negative coefficient of PaCO_2_, an explanatory model excluding pH was fitted, where PaCO_2_ showed a positive association with VIS, consistent with the physiologically expected harmful effect of respiratory acidosis. However, when pH was added to the model, the pH term became the dominant predictor and the PaCO₂ term inverted. This confirms that the haemodynamic benefit of ECMO is mediated through the correction of the overall acid-base status (pH) rather than PaCO₂ per se. We interpret the negative PaCO₂ coefficient in the final model because of the model structure: when holding pH constant, a higher PaCO₂ mathematically necessitates a higher serum bicarbonate level (i.e., metabolic compensation) due to their interdependence in the Henderson-Hasselbalch equation. Serum bicarbonate was excluded from the multivariable model due to this structural multicollinearity. Instead, we included serum lactate (which showed low collinearity with pH and PaCO₂) to serve as an independent marker of metabolic status. We excluded CRP from the multivariable model due to the observed dissociation between CRP and hemodynamic trajectories (Table 2). While vasopressor requirements decreased significantly, median CRP levels increased from 16 mg/dL on Day 0–22 mg/dL on Day 1. This paradoxical rise could reflect the immediate pro-inflammatory response to the extracorporeal circuit [22] and the slower pharmacokinetics of CRP, confirming that the rapid haemodynamic stabilisation occurred independently of systemic inflammation resolution. It is important to stress that despite the importance of correcting respiratory acidosis, CO_2_ clearance must be reduced gradually over several hours, to prevent dangerous rapid changes in cerebral perfusion [23].

To address the potential for regression-to-the-mean, we analysed the subgroup of patients with low baseline noradrenaline requirements (≤0.05 µg/kg/min). In this subgroup, no significant association was found between arterial pH and VIS. We interpret this as a physiological 'floor effect': in patients with minimal vasopressor requirements, haemodynamic stability precludes further significant reduction in VIS regardless of acid-base correction. In contrast, the association remained strong and significant in the high-severity subgroup (noradrenaline ≥0.2 µg/kg/min), where the physiological need for vasopressor support was high. This dissociation confirms that the observed benefit of respiratory acidosis correction is likely a clinically relevant effect specific to patients with significant haemodynamic compromise, rather than a statistical artifact.

Of note, despite haemodynamic impairment before ECMO start, few patients in our cohort received inotropic support. From a clinical perspective, inotropic support with dobutamine is typically considered a second-line or adjunctive treatment, and likely, the rapid progression to ECMO may have obviated a trial of inotropes. As our study lacks detailed echocardiographic or pulmonary artery catheter measurements, we cannot determine the true prevalence and degree of RV dysfunction.

A noteworthy observation from our model was the lack of an independent effect of PaO_2_ on VIS. Previous research demonstrated that increasing the mixed venous oxygen saturation (SvO_2_) via higher V-V ECMO blood flow could significantly improve pulmonary vascular resistance and RV function, despite minimal changes in pH [11]. In our cohort, the minimal change in mean PaO_2_ from Day 0 to Day 1 (Table 2) suggests that ECMO blood flow was titrated primarily to achieve normoxaemia. However, regardless of the PaO_2_ resulting from V-V ECMO and native lung function, the admixture of oxygenated blood before the pulmonary circulation, thus raising the SvO_2_, could very well have contributed to the haemodynamic improvements. Due to unavailability of pulmonary artery catheter measurements, the exact differentiation between the effects of higher SvO_2_ vs. acidosis correction could unfortunately not be studied.

Of particular interest was the observation that mean airway pressure (P_aw_) decreased only modestly after V-V ECMO initiation, which may have led to an underestimation of its full potential for haemodynamic improvement. This is partly explained by the fact that PEEP remained unchanged (Table 2). Maintaining adequate PEEP is crucial to prevent alveolar collapse and the subsequent rise in PVR associated with the lower limb of the PVR-inflation curve [14].

However, this observation also suggests a potential pitfall in clinical practice: while clinicians may reduce the set driving pressure and respiratory rate, the resulting decrease in P_aw_ can be less than anticipated if the I:E ratio is not concurrently adjusted. A lower respiratory rate inherently prolongs the absolute inspiratory time at a fixed I:E ratio, so that the net decrease in P_aw_ becomes disproportionate to the decrease in driving pressure. This raises the question of whether a more deliberate strategy of adjusting the I:E ratio to shorten inspiratory time could yield more significant and immediate haemodynamic benefits. However, we must also acknowledge a key limitation: our analysis relied on P_aw_, not the more physiologically precise oesophageal or pleural pressure. As P_aw_ is only partially transmitted to the pleural space, especially in patients with the low-compliance lungs characteristic of severe ARDS [5], the true effect of our observed ventilator changes on RV afterload can only be partially extrapolated.

Our study builds upon the findings of a similar recent analysis by Odish et al., though our methodology differed in several key aspects [15]. While we used VIS as our primary outcome, the Odish study used the “norepinephrine equivalent” (NEE) dose. Despite this difference and the caveat that their score excludes dobutamine, our cohorts had comparable baseline vasopressor requirements (median NEE 0.14 µg/kg/min or mean 0.28 µg/kg/min in our study vs. mean 0.23 µg/kg/min in the Odish study). However, several important distinctions in study design and patient populations exist. For instance, our study included a longer observation period (from Day -2 to Day 3 vs. immediately before to 24 h after cannulation), analysed mean 24 -h medication doses rather than spot measurements, had no patients receiving adrenaline, and had a higher proportion of female patients (37% vs. 25.9%). The six-day observation window (Day -2 to Day +3) in our study was selected to capture the pre-ECMO physiological trajectory and to confirm the sustainability of the haemodynamic improvement beyond the immediate procedural period. Despite these differences, our core findings were consistent: vasopressor requirements decreased significantly after V-V ECMO initiation, and this improvement was strongly associated with the correction of arterial pH.

These findings also hold implications for the choice of ECMO configuration. While severe haemodynamic compromise often triggers consideration for venoarterial or veno-arteriovenous support, our data demonstrate that substantial haemodynamic stabilisation is achievable with V-V ECMO alone. Consequently, high vasopressor requirements should not automatically necessitate venoarterial ECMO if the circulatory failure is primarily driven by respiratory acidosis and RV afterload, rather than primary myocardial dysfunction.

A key strength of our analysis was the inclusion of fluid balance and sedative agents as potential haemodynamic confounders. We found a trend towards a positive association between cumulative fluid balance and VIS; however, a sensitivity analysis using a time-lagged covariate suggested that fluid administration was more likely a reaction to hemodynamic instability rather than a contributor. Regarding sedative agents, our analysis revealed a positive association between higher propofol dosage and increased VIS that did not reach statistical significance.

General limitations of our study are inherent to its retrospective design. Due to the lack of a control group, our study cannot answer how vasopressor requirements would have evolved without V-V ECMO cannulation. Furthermore, as a single-centre study using data collected over several years, our findings may be influenced by local treatment protocols, patient populations and potential changes over time, which could limit the generalisability of our results to other institutions with different practices. Also, while our multivariable model adjusted for several key physiological variables, the potential for unmeasured confounders remains. To account for measurements being taken at different exact times during routine clinical care, covariates were aggregated into daily means. A necessary consequence of this approach is that the impact of intra-day parameter fluctuations could not be assessed.

Interpretation

In our study, the initiation of V-V ECMO was associated with a significant improvement in haemodynamic status, evidenced by a marked decrease in vasopressor requirements. Our multivariable analysis demonstrates that this benefit is directly attributable to the important therapeutic effect of correcting severe respiratory acidosis. Therefore, the ability of V-V ECMO to restore acid-base balance should be considered as an important means for haemodynamic stabilisation in patients with severe respiratory failure.

## CRediT authorship contribution statement

BN was involved in study design, data collection, data analysis, data interpretation and writing. NB, OR, PS, and AH were involved in data collection, data interpretation and manuscript preparation. TS was involved in study design, data collection, data analysis, data interpretation and manuscript preparation. All authors helped to revise the draft of the manuscript. All authors read and approved the final manuscript.

## Consent for publication

Not applicable.

## Ethics approval and consent to participate

The protocol was approved by the institutional review board of the Medical University of Vienna (Ref. EK 1630/2024). Due to the retrospective design of the study and the collection of data from routine care, the requirement of individual patient consent was waived.

## Declaration of Generative AI and AI-assisted technologies in the writing process

During the preparation of this work, the authors used Gemini (Google) and DeepL for assistance in refining the language and grammar of this manuscript to improve readability. After using these tools/services, the authors reviewed and edited the content as needed and take full responsibility for the content of the publication.

## Funding

No third-party funding was received.

The study was sponsored by the Medical University of Vienna. No other sources of funding were used.

## Availability of data and material

The datasets used and/or analysed during the current study are available from the corresponding author on reasonable request.

## Declaration of competing interest

TS: speaker fees: Getinge, Xenios. PS: speaker fees: Getinge, Fresenius Medical. AH: speaker fees: Getinge. All other authors have no conflicts of interest to disclose.
