# Supplementary material for: Vasopressor Requirements after Initiation of Venovenous Extracorporeal Membrane Oxygenation in Patients with Severe Respiratory Failure
Source: Ann Intensive Care. 2026 Jan 16;16:100023. doi: 10.1016/j.aicoj.2025.100023 (PMC12934440; doi:10.1016/j.aicoj.2025.100023)
Supplement: Supplementary file 1 [file mmc1.docx]

e-Table 1. Number and Percentage of Missing Observations for Covariables of the Mixed-Effects Model.

| Variable | Day -2 (N=28) | Day -1 (N=39) | Day 0 (N=107) | Day 1 (N=107) | Day 2 (N=104) | Day 3 (N=101) |
| --- | --- | --- | --- | --- | --- | --- |
| VIS | 0 (0%) | 0 (0%) | 0 (0%) | 0 (0%) | 0 (0%) | 0 (0%) |
| Daily Net Fluid Balance | 0 (0%) | 1 (2.6%) | 8 (7.5%) | 1 (0.9%) | 0 (0%) | 0 (0%) |
| Mean Airway Pressure | 2 (7.1%) | 1 (2.6%) | 3 (2.8%) | 2 (1.9%) | 2 (1.9%) | 2 (2%) |
| Arterial pH | 0 (0%) | 0 (0%) | 0 (0%) | 0 (0%) | 0 (0%) | 0 (0%) |
| PaO2 | 0 (0%) | 0 (0%) | 1 (0.9%) | 0 (0%) | 0 (0%) | 0 (0%) |
| PaCO2 | 0 (0%) | 0 (0%) | 0 (0%) | 0 (0%) | 0 (0%) | 0 (0%) |
| Propofol Dose | 0 (0%) | 0 (0%) | 0 (0%) | 0 (0%) | 0 (0%) | 0 (0%) |
| Lactate | 0 (0%) | 0 (0%) | 0 (0%) | 0 (0%) | 0 (0%) | 0 (0%) |
| Values are presented as n (%). Percentages are calculated based on the total number of patients (N) present in the study unit on that specific day relative to ECMO initiation. | | | | | | |
