# Supplementary material for: Vasopressor Requirements after Initiation of Venovenous Extracorporeal Membrane Oxygenation in Patients with Severe Respiratory Failure
Source: Ann Intensive Care. 2026 Jan 16;16:100023. doi: 10.1016/j.aicoj.2025.100023 (PMC12934440; doi:10.1016/j.aicoj.2025.100023)
Supplement: Supplementary file 2 [file mmc2.docx]

e-Table 2. Descriptive Comparison of Additional Clinical Parameters between „Day 0“ (24h before ECMO start) and „Day 1” (24h after ECMO start).

| **Characteristic** | **Day 0** N = 107*^1^* | **Day 1** N = 107*^1^* | **p-value***^2^* |
| --- | --- | --- | --- |
| Lowest PaO₂ (mmHg) | 61 (55, 68) | 63 (56, 77) | 0.11 |
| Highest PaO₂ (mmHg) | 114 (82, 145) | 115 (100, 132) | >0.9 |
| Lowest PaCO₂ (mmHg) | 53 (44, 65) | 44 (39, 50) | <0.001 |
| Highest PaCO₂ (mmHg) | 77 (65, 91) | 66 (58, 77) | <0.001 |
| Mean Systolic Arterial Blood Pressure (mmHg) | 120 (112, 127) | 122 (116, 130) | 0.061 |
| Mean Diastolic Arterial Blood Pressure (mmHg) | 59 (55, 65) | 62 (57, 66) | 0.030 |
| Highest Lactate (mmol/l) | 1.50 (1.10, 2.90) | 2.00 (1.30, 2.90) | <0.001 |
| Lowest pH | 7.25 (7.17, 7.31) | 7.31 (7.25, 7.36) | <0.001 |
| Highest pH | 7.38 (7.32, 7.43) | 7.47 (7.43, 7.50) | <0.001 |
| Mean Dobutamine Dose (µg/kg/min) | 2.56 (2.47, 7.53)  (N=7) | 4.08 (2.50, 5.88)  (N=11) | 0.8 |
| Mean Vasopressin Dose (U/hr) | 2.00 (1.50, 2.38)  (N=15) | 1.54 (0.83, 2.00)  (N=18) | 0.10 |
| Mean Remifentanil Dose (µg/kg/min) | 0.17 (0.11, 0.24)  (N=52) | 0.18 (0.14, 0.28)  (N=45) | 0.004 |
| Mean Sufentanil Dose (µg/kg/hr) | 2.44 (1.83, 3.20)  (N=59) | 2.45 (2.00, 3.14)  (N=64) | 0.088 |
| Mean Midazolam Dose (mg/kg/hr) | 0.2 (0.1, 0.4)  (N=53) | 0.2 (0.1, 0.4)  (N=65) | 0.13 |
| Mean Dexmedetomidine Dose (µg/kg/hr) | 0.70 (0.28, 1.11)  (N=4) | 1. (0.70, 1.00)   (N=9) | >0.9 |
| Mean FiO₂ (%) | 82 (70, 92) | 49 (40, 62) | <0.001 |
| Mean I:E Ratio (Expiratory part) | 1.99 (1.80, 2.00) | 2.00 (1.91, 2.00) | 0.3 |
| Mean ECMO Blood Flow (l/min) |  | 3.51 (2.75, 4.07) |  |
| Mean ECMO RPM (1/min) |  | 2,824 (2,425, 3,319) |  |
| Mean ECMO Gas Flow (l/min) |  | 3 (2, 4) |  |
| *^1^* Median (Q1, Q3) | | | |
| *^2^* Wilcoxon signed rank test with continuity correction; Wilcoxon signed rank exact test  FiO₂, fraction of inspired oxygen; I:E, inspiratory to expiratory; PaCO₂, partial pressure of arterial carbon dioxide; PaO₂, partial pressure of arterial oxygen. | | | |
