# Supplementary material for: Vasopressor Requirements after Initiation of Venovenous Extracorporeal Membrane Oxygenation in Patients with Severe Respiratory Failure
Source: Ann Intensive Care. 2026 Jan 16;16:100023. doi: 10.1016/j.aicoj.2025.100023 (PMC12934440; doi:10.1016/j.aicoj.2025.100023)
Supplement: Supplementary file 3 [file mmc3.docx]

e-Table 3. Linear Mixed-Effects Model of Covariates Associated with Vasoactive-Inotropic Score (VIS) from Day -2 to Day 3

| **Characteristic** | **Beta** | **95% CI** | **p-value** |
| --- | --- | --- | --- |
| (Intercept) | 12 | 6.7, 18 | <0.001 |
| Daily Net Fluid Balance (standardised) | 2.0 | 0.57, 3.4 | 0.006 |
| Mean Airway Pressure (standardised) | 0.87 | -0.96, 2.7 | 0.4 |
| Mean Arterial pH (standardised) | -6.8 | -9.3, -4.3 | <0.001 |
| Mean Arterial PaO₂ (standardised) | 0.28 | -1.2, 1.8 | 0.7 |
| Mean Arterial PaCO₂ (standardised) | -3.3 | -5.6, -0.90 | 0.007 |
| Mean Propofol Dose (standardised) | 0.92 | -1.0, 2.9 | 0.3 |
| Mean Lactate (standardised) | 10 | 8.4, 13 | <0.001 |
| Renal Replacement Therapy (Active) | -2.8 | -8.8, 3.2 | 0.4 |
| CI, Confidence Interval; PaCO₂, partial pressure of arterial carbon dioxide; PaO₂, partial pressure of arterial oxygen  Note: Covariates were standardised (z-score scaled) to a mean of 0 and a standard deviation of 1 to facilitate the comparison of effects. Time, which was included as a factor in the model, is not shown here for clarity. Effects over time are presented by estimated marginal means. | | | |
