# Supplementary material for: Vasopressor Requirements after Initiation of Venovenous Extracorporeal Membrane Oxygenation in Patients with Severe Respiratory Failure
Source: Ann Intensive Care. 2026 Jan 16;16:100023. doi: 10.1016/j.aicoj.2025.100023 (PMC12934440; doi:10.1016/j.aicoj.2025.100023)
Supplement: Supplementary file 4 [file mmc4.docx]

e-Table 4. Linear Mixed-Effects Model of Covariates Associated with Vasoactive-Inotropic Score (VIS) from Day -2 to Day 3 Using a Time-Lagged Covariate for Fluid Balance

| **Characteristic** | **Beta** | **95% CI** | **p-value** |
| --- | --- | --- | --- |
| (Intercept) | 9.7 | 4.2, 15 | <0.001 |
| Daily Net Fluid Balance From Previous Day (standardised) | 1.5 | -0.09, 3.1 | 0.063 |
| Mean Airway Pressure (standardised) | 0.38 | -1.8, 2.5 | 0.7 |
| Mean Arterial pH (standardised) | -5.9 | -9.0, -2.7 | <0.001 |
| Mean Arterial PaO₂ (standardised) | -0.72 | -2.8, 1.3 | 0.5 |
| Mean Arterial PaCO₂ (standardised) | 0.49 | -2.5, 3.5 | 0.7 |
| Mean Propofol Dose (standardised) | 2.4 | 0.30, 4.5 | 0.026 |
| CI, Confidence Interval; PaCO₂, partial pressure of arterial carbon dioxide; PaO₂, partial pressure of arterial oxygen  Note: Covariates were standardised (z-score scaled) to a mean of 0 and a standard deviation of 1 to facilitate the comparison of effects. Time, which was included as a factor in the model, is not shown here for clarity. Fluid balance was included as a ‘lagged variable’, to assess the impact of the fluid balance from the previous day on VIS. | | | |
