# Supplementary material for: Vasopressor Requirements after Initiation of Venovenous Extracorporeal Membrane Oxygenation in Patients with Severe Respiratory Failure
Source: Ann Intensive Care. 2026 Jan 16;16:100023. doi: 10.1016/j.aicoj.2025.100023 (PMC12934440; doi:10.1016/j.aicoj.2025.100023)
Supplement: Supplementary file 5 [file mmc5.docx]

e-Table 5. Baseline Characteristics of the Subgroup of Patients requiring a Mean Noradrenaline Dose of ≥0.2 µg/kg/min on Day 0

| **Characteristic** | **N = 40***^1^* |
| --- | --- |
| Sex |  |
| Female | 13 (33%) |
| Male | 27 (68%) |
| Age at admission (years) | 47 (35, 59) |
| Height (cm) | 173 (167, 180) |
| Weight (kg) | 78 (70, 90) |
| Body Mass Index | 26 (23, 30) |
| SOFA (Sequential Organ Failure Assessment) score at ICU Admission | 11.0 (9.0, 13.0) |
| SAPS III (Simplified Acute Physiology Score III) at ICU admission | 72 (66, 81) |
| Lowest PaO₂/FiO₂ ratio on day of ECMO start | 70 (56, 95) |
| Lowest pH on day of ECMO start | 7.20 (7.13, 7.29) |
| Lowest PaO₂ on day of ECMO start (mmHg) | 62 (54, 73) |
| Highest PaCO₂ on day of ECMO start (mmHg) | 77 (63, 98) |
| Mean Peak Inspiratory Pressure on day of ECMO start (mbar) | 31.7 (28.0, 34.0) |
| Mean PEEP on day of ECMO start (mbar) | 12.3 (10.0, 15.3) |
| Mean Respiratory Rate on day of ECMO start (breaths/min) | 19.8 (17.0, 22.3) |
| Mean Airway Pressure on day of ECMO start (mbar) | 19.2 (15.7, 21.8) |
| NT-proBNP at ECMO start (pg/ml) | 1,315 (487, 5,827) |
| Noradrenaline at ECMO start | 40 (100%) |
| Mean Noradrenaline Dose on day of ECMO start (µg/kg/min) | 0.37 (0.27, 0.86) |
| Dobutamine at ECMO start | 5 (13%) |
| Vasopressin at ECMO start | 14 (35%) |
| Mean Vasoactive-Inotropic Score on day of ECMO start | 35 (26, 98) |
| Highest Lactate on day of ECMO start (mmol/l) | 3.1 (1.5, 7.1) |
| Mean Lactate on day of ECMO start (mmol/l) | 2.36 (1.40, 5.09) |
| Cause of respiratory failure |  |
| Influenza | 8 (20%) |
| Other infectious pneumonia | 8 (20%) |
| COVID 19 | 7 (18%) |
| COPD/Asthma | 3 (7.5%) |
| Exacerbation of other chronic respiratory disease | 3 (7.5%) |
| Other | 11 (27.5%) |
| Duration of ECMO support (days) | 12 days (7 days, 22 days) |
| Renal Replacement Therapy During ICU Stay | 17 (43%) |
| ICU Survival | 23 (58%) |
| Hospital Survival | 22 (56%) |
| *^1^* n (%); Median (Q1, Q3)  ECMO, extracorporeal membrane oxygenation; FiO₂, fraction of inspired oxygen; ICU, intensive care unit; IQR, interquartile range; NT-proBNP, N-terminal pro–B-type natriuretic peptide; PaCO₂, partial pressure of arterial carbon dioxide; PaO₂, partial pressure of arterial oxygen; PEEP, positive end-expiratory pressure; SAPS III, Simplified Acute Physiology Score III; SOFA, Sequential Organ Failure Assessment; VIS, Vasoactive-Inotropic Score. | |
