# Supplementary material for: Vasopressor Requirements after Initiation of Venovenous Extracorporeal Membrane Oxygenation in Patients with Severe Respiratory Failure
Source: Ann Intensive Care. 2026 Jan 16;16:100023. doi: 10.1016/j.aicoj.2025.100023 (PMC12934440; doi:10.1016/j.aicoj.2025.100023)
Supplement: Supplementary file 6 [file mmc6.docx]

e-Table 6. Changes between „Day 0“ (24h before ECMO start) and „Day 1” (24h after ECMO start) in the Subgroup of Patients requiring a Mean Noradrenaline Dose of ≥0.2 µg/kg/min on Day 0

| **Characteristic** | **Day 0** N = 40*^1^* | **Day 1** N = 40*^1^* | **p-value***^2^* |
| --- | --- | --- | --- |
| Mean Vasoactive-Inotropic Score | 35 (26, 98) | 27 (17, 61) | <0.001 |
| Mean Noradrenaline Dose (µg/kg/min) | 0.37 (0.27, 0.86) | 0.24 (0.14, 0.51) | <0.001 |
| Mean Dobutamine Dose (µg/kg/min) | 6.94 (2.51, 7.53)  (N=5) | 4.33 (2.50, 5.88)  (N=10) | 0.8 |
| Mean Vasopressin Dose (U/hr) | 2.00 (1.50, 2.38)  (N=14) | 1.58 (1.00, 2.00)  (N=17) | 0.10 |
| Cumulative Fluid Balance (Day 0-1) (ml) |  | 2,904 (1,726, 5,779) |  |
| Mean Systolic Arterial Blood Pressure (mmHg) | 119 (111, 123) | 124 (117, 133) | 0.008 |
| Mean Diastolic Arterial Blood Pressure (mmHg) | 60 (58, 66) | 64 (58, 69) | 0.3 |
| Mean Mean Arterial Blood Pressure (mmHg) | 79 (72, 83) | 80 (77, 87) | 0.026 |
| Mean Heart Rate (bpm) | 108 (96, 120) | 93 (82, 102) | <0.001 |
| Mean SpO2 (%) | 93.4 (89.3, 95.7) | 97.2 (95.2, 98.3) | <0.001 |
| Highest Lactate on day of ECMO start (mmol/l) | 3.1 (1.5, 7.1) | 3.1 (1.9, 8.7) | <0.001 |
| Mean Lactate on day of ECMO start (mmol/l) | 2.4 (1.4, 5.1) | 2.1 (1.3, 6.2) | 0.12 |
| Mean NT-proBNP (pg/ml) | 1,315 (487, 5,827) | 2,756 (1,174, 7,267) | 0.4 |
| Mean Mean Airway Pressure (mbar) | 19.2 (15.7, 21.8) | 16.3 (13.7, 20.7) | <0.001 |
| Mean PEEP (mbar) | 12.3 (10.0, 15.3) | 11.2 (9.5, 15.0) | 0.3 |
| Mean Peak Inspiratory Pressure (mbar) | 31.7 (28.0, 34.0) | 25.8 (23.0, 29.1) | <0.001 |
| Mean Tidal Volume (ml) | 448 (337, 497) | 292 (225, 364) | <0.001 |
| Mean Respiratory Rate (breaths/min) | 19.8 (17.0, 22.3) | 13.6 (12.1, 15.5) | <0.001 |
| Mean I:E Ratio (Expiratory part) | 1.99 (1.69, 2.00) | 2.00 (1.91, 2.00) | 0.11 |
| Mean FiO₂ (%) | 87 (76, 95) | 49 (40, 67) | <0.001 |
| Lowest PaO₂ (mmHg) | 62 (54, 73) | 68 (58, 79) | 0.2 |
| Highest PaO₂ (mmHg) | 118 (83, 152) | 120 (102, 156) | 0.6 |
| Mean PaO₂ (mmHg) | 82 (73, 99) | 88 (75, 102) | 0.6 |
| Lowest PaCO₂ (mmHg) | 50 (39, 66) | 40 (35, 48) | <0.001 |
| Highest PaCO₂ (mmHg) | 77 (63, 98) | 61 (53, 68) | <0.001 |
| Mean PaCO₂ (mmHg) | 63 (53, 77) | 49 (43, 55) | <0.001 |
| Lowest pH | 7.20 (7.13, 7.29) | 7.29 (7.22, 7.33) | <0.001 |
| Highest pH | 7.33 (7.28, 7.40) | 7.47 (7.41, 7.51) | <0.001 |
| Mean pH | 7.26 (7.21, 7.35) | 7.38 (7.33, 7.43) | <0.001 |
| Mean Propofol Dose (mg/kg/hr) | 2 (2, 4)  (N=32) | 2 (2, 3)  (N=30) | 0.2 |
| Mean Remifentanil Dose (µg/kg/min) | 0.17 (0.11, 0.23)  (N=28) | 0.18 (0.14, 0.28)  (N=27) | 0.025 |
| Mean Sufentanil Dose (µg/kg/hr) | 2.60 (1.50, 3.43)  (N=13) | 3.12 (2.36, 4.08)  (N=14) | 0.092 |
| Mean Midazolam Dose (mg/kg/hr) | 0.2 (0.2, 0.4)  (N=16) | 0.3 (0.1, 0.4)  (N=22) | 0.6 |
| Mean Dexmedetomidine Dose (µg/kg/hr) | 1. (1.00, 1.00)   (N=1) | 0.93 (0.68, 1.00)  (N=4) | >0.9 |
| Mean ECMO Blood Flow (l/min) |  | 3.74 (2.98, 4.10) |  |
| Mean ECMO RPM (1/min) |  | 2,842 (2,352, 3,135) |  |
| Mean ECMO Gas Flow (l/min) |  | 3 (2, 5) |  |
| Renal Replacement Therapy | 4 (10%) | 8 (20%) | 0.2 |
| *^1^* Median (Q1, Q3) | | | |
| *^2^* Wilcoxon signed rank test with continuity correction; Wilcoxon signed rank exact test  N, number of patients; NT-proBNP, N-terminal pro–B-type natriuretic peptide; PaCO₂, partial pressure of arterial carbon dioxide; PaO₂, partial pressure of arterial oxygen; PEEP, positive end-expiratory pressure | | | |
