# Supplementary material for: Vasopressor Requirements after Initiation of Venovenous Extracorporeal Membrane Oxygenation in Patients with Severe Respiratory Failure
Source: Ann Intensive Care. 2026 Jan 16;16:100023. doi: 10.1016/j.aicoj.2025.100023 (PMC12934440; doi:10.1016/j.aicoj.2025.100023)
Supplement: Supplementary file 7 [file mmc7.docx]

e-Table 7. Linear Mixed-Effects Model of Covariates Associated with Vasoactive-Inotropic Score (VIS) from Day -2 to Day 3 for the Subgroup of Patients requiring a Mean Noradrenaline Dose of ≥0.2 µg/kg/min on Day 0

| **Characteristic** | **Beta** | **95% CI** | **p-value** |
| --- | --- | --- | --- |
| (Intercept) | 949 | 384, 1,515 | 0.001 |
| Daily Net Fluid Balance (per +1,000 ml) | 0.57 | -0.88, 2.0 | 0.4 |
| Mean Airway Pressure (per +1 mbar) | -0.05 | -1.1, 1.0 | >0.9 |
| Mean Arterial pH (per +0.1 unit) | -12 | -20, -5.0 | 0.001 |
| Mean PaO₂ (per +1 mmHg) | -0.05 | -0.21, 0.11 | 0.6 |
| Mean PaCO₂ (per +10 mmHg) | -5.3 | -9.3, -1.4 | 0.009 |
| Mean Propofol Dose (mg/kg/h) | 0.04 | -0.03, 0.11 | 0.3 |
| Mean Lactate (mmol/l) | 3.1 | 1.5, 4.6 | <0.001 |
| Renal Replacement Therapy (Active) | -7.8 | -20, 4.7 | 0.2 |
| CI, Confidence Interval; PaCO₂, partial pressure of arterial carbon dioxide; PaO₂, partial pressure of arterial oxygen  Conditional R²: 0.73; Marginal R²: 0.58  Note: Estimates represent the change in VIS associated with the specified unit increase in each predictor. Time (study day), which was included as a factor in the model to account for temporal trends, is not shown here for clarity. | | | |
