# Supplementary material for: Vasopressor Requirements after Initiation of Venovenous Extracorporeal Membrane Oxygenation in Patients with Severe Respiratory Failure
Source: Ann Intensive Care. 2026 Jan 16;16:100023. doi: 10.1016/j.aicoj.2025.100023 (PMC12934440; doi:10.1016/j.aicoj.2025.100023)
Supplement: Supplementary file 8 [file mmc8.docx]

e-Table 8. Linear Mixed-Effects Model of Covariates Associated with Vasoactive-Inotropic Score (VIS) from Day -2 to Day 3 for the Subgroup of Patients requiring a Mean Noradrenaline Dose of ≤0.05 µg/kg/min on Day 0

| **Characteristic** | **Beta** | **95% CI** | **p-value** |
| --- | --- | --- | --- |
| (Intercept) | 32 | -92, 156 | 0.6 |
| Daily Net Fluid Balance (per +1,000 ml) | -0.24 | -0.58, 0.09 | 0.2 |
| Mean Airway Pressure (per +1 mbar) | -0.10 | -0.30, 0.10 | 0.3 |
| Mean Arterial pH (per +0.1 unit) | -0.41 | -2.0, 1.2 | 0.6 |
| Mean PaO₂ (per +1 mmHg) | 0.00 | -0.03, 0.02 | 0.7 |
| Mean PaCO₂ (per +10 mmHg) | 0.21 | -0.59, 1.0 | 0.6 |
| Mean Propofol Dose (mg/kg/h) | 0.00 | -0.01, 0.01 | >0.9 |
| Mean Lactate (mmol/l) | 0.75 | -0.65, 2.1 | 0.3 |
| CI, Confidence Interval; PaCO₂, partial pressure of arterial carbon dioxide; PaO₂, partial pressure of arterial oxygen  Conditional R²: 0.68; Marginal R²: 0.1  Note: Estimates represent the change in VIS associated with the specified unit increase in each predictor. Time (study day), which was included as a factor in the model to account for temporal trends, is not shown here for clarity. No patients in this subgroup received renal replacement therapy, so that this covariable was not included. | | | |
