# Supplementary figures and images for: Vasopressor Requirements after Initiation of Venovenous Extracorporeal Membrane Oxygenation in Patients with Severe Respiratory Failure
Source: Ann Intensive Care. 2026 Jan 16;16:100023. doi: 10.1016/j.aicoj.2025.100023 (PMC12934440; doi:10.1016/j.aicoj.2025.100023)

# Collinearity

High collinearity (VIF) may inflate parameter uncertainty

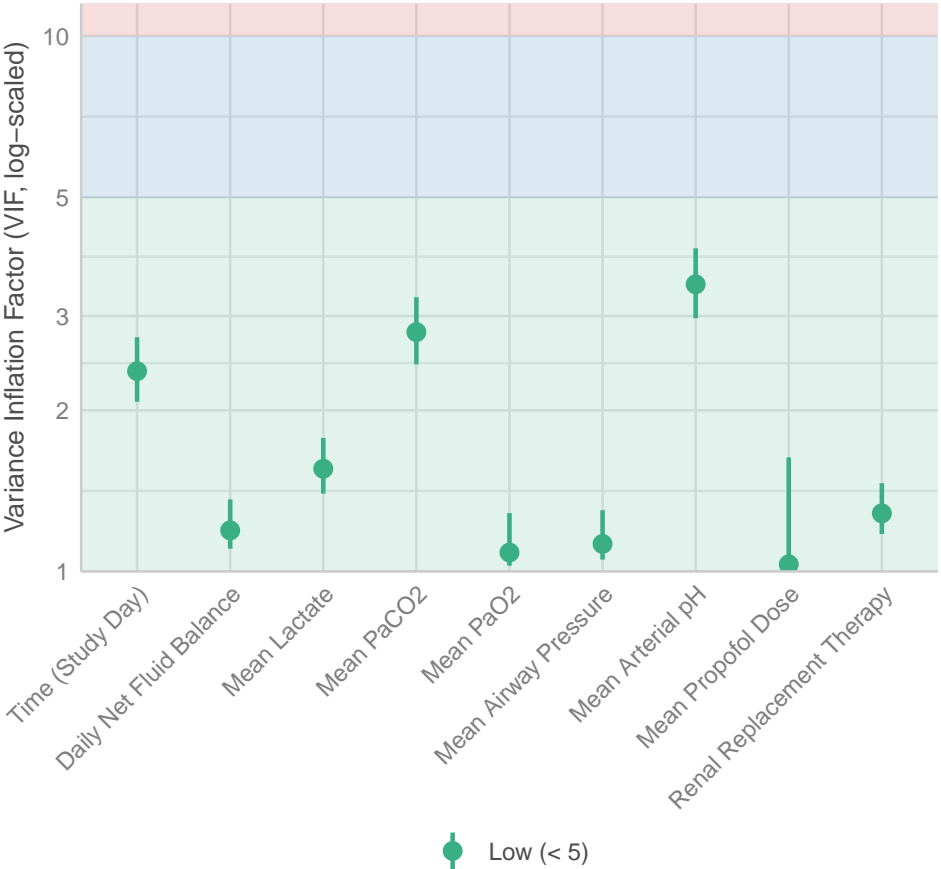

Supplement: Supplementary file 9 [file mmc9.pdf]

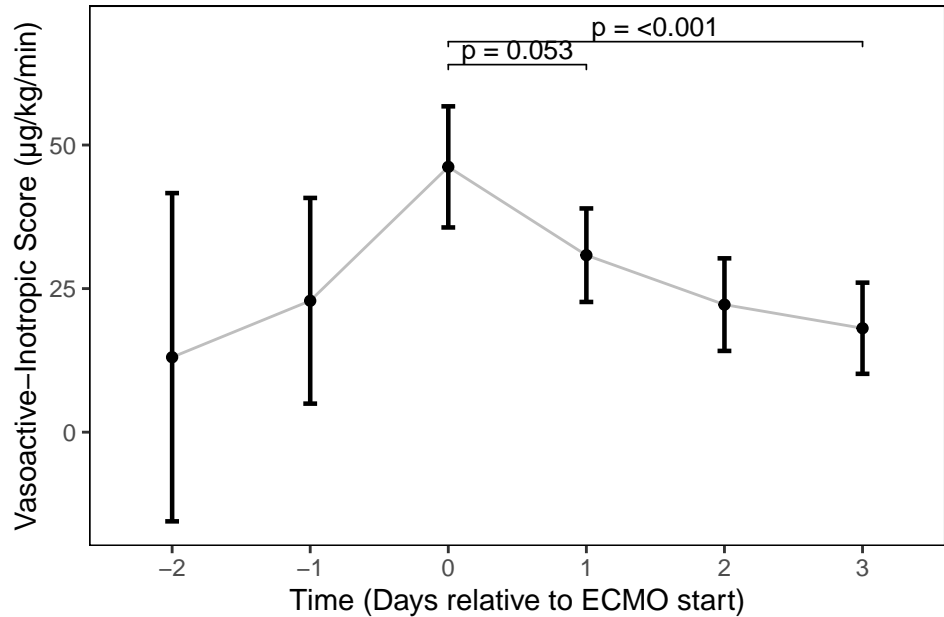

Supplement: Supplementary file 10 [file mmc10.pdf]
